# The Increasing Interest of ANAMMOX Research in China: Bacteria, Process Development, and Application

**DOI:** 10.1155/2013/134914

**Published:** 2013-12-05

**Authors:** Mohammad Ali, Li-Yuan Chai, Chong-Jian Tang, Ping Zheng, Xiao-Bo Min, Zhi-Hui Yang, Lei Xiong, Yu-Xia Song

**Affiliations:** ^1^Department of Environmental Engineering, School of Metallurgy and Environment, Central South University, Lushan South Road 932, Changsha, Hunan 410083, China; ^2^National Engineering Research Centre for Control and Treatment of Heavy Metal Pollution, Changsha 410083, China; ^3^Department of Environmental Engineering, Zhejiang University, Hangzhou 310058, China

## Abstract

Nitrogen pollution created severe environmental problems and increasingly has become an important issue in China. Since the first discovery of ANAMMOX in the early 1990s, this related technology has become a promising as well as sustainable bioprocess for treating strong nitrogenous wastewater. Many Chinese research groups have concentrated their efforts on the ANAMMOX research including bacteria, process development, and application during the past 20 years. A series of new and outstanding outcomes including the discovery of new ANAMMOX bacterial species (Brocadia sinica), sulfate-dependent ANAMMOX bacteria (Anammoxoglobus sulfate and *Bacillus benzoevorans*), and the highest nitrogen removal performance (74.3–76.7 kg-N/m^3^/d) in lab scale granule-based UASB reactors around the world were achieved. The characteristics, structure, packing pattern and floatation mechanism of the high-rate ANAMMOX granules in ANAMMOX reactors were also carefully illustrated by native researchers. Nowadays, some pilot and full-scale ANAMMOX reactors were constructed to treat different types of ammonium-rich wastewater including monosodium glutamate wastewater, pharmaceutical wastewater, and leachate. The prime objective of the present review is to elucidate the ongoing ANAMMOX research in China from lab scale to full scale applications, comparative analysis, and evaluation of significant findings and to set a design to usher ANAMMOX research in culmination.

## 1. Introduction

Anaerobic ammonia oxidation (ANAMMOX) process is a novel and promising biological nitrogen removal biotechnology that has been successfully applied from the beginning of this century [1–3]. ANAMMOX bacteria convert ammonium directly to nitrogen gas with nitrite as the electron acceptor under anoxic conditions. From the discovery of the ANAMMOX process in mid 1990s [1], it has been regarded as a cost-effective and environment-friendly way to treat wastewater containing high ammonium concentrations [4].

By smart application of ANAMMOX in municipal treatment, wastewater treatment plants could be converted from energy-consuming into energy-producing systems [5]. It becomes a hot topic in the fields of microbiology and environmental science and engineering due to its merits of effective removal of both ammonium and nitrite under anaerobic conditions with high removal rate, little sludge production, and low operational cost [5–8].

The drawback of this new and cost-effective process is the low growth rate of involved bacteria (0.0027 h^−1^ reported by Strous et al. [2]; 0.016 h^−1^ reported by Isaka et al. [9]). So, the ANAMMOX reactors are often operated at a long solids retention time (SRT) in order to accumulate the necessary biomass in the system [2, 10, 11]. Till now 5 genera of ANAMMOX bacteria including 13 species [12] have been identified and, among these, *Candidatus *Brocadia sinica has been discovered in China [13]. On the other hand, the existence of sulfate-dependent anaerobic ammonium oxidation has been recognized so far, but the involved microorganisms have been isolated infrequently. Recently new species of sulfate-dependent ANAMMOX bacteria Anammoxoglobus sulfate [14] and *Bacillus benzoevorans* [15] have been discovered in China which are the functional community to remove ammonium and sulfate simultaneously.

High rate is one of the prime objectives for ANAMMOX process. Within the last decades some specialized reactor systems such as sequencing batch reactor (SBR) [2, 16–19], rotating biological contactor (RBC) [18], trickling filter [19], UBF reactor [20], granular sludge bed reactor [21], and membrane bioreactor [3, 22] have been introduced in both laboratory and full scale to obtain high removal rate, and finally noticed that these reactors played an important role in securing high rate performance for ammonium and nitrite removal. The NRR of conventional nitrogen removal biotechnologies was less than 0.5 kg-N/m^3^/d [20] while, for ANAMMOX process, it was higher than 5 kg-N/m^3^/d as obtained by a number of researchers using different reactors such as upflow biofilter, upflow anaerobic sludge blanket (UASB) reactor, and gas-lift reactor [3, 23–26].

Nitrogen pollution (N-pollution) causes serious environmental problems. N-pollution not only hampers the sustainable development of agriculture, fishery, tourism, and so forth but also threatens the living environment of human beings. According to the report of “the state of the environment in China (2009),” the ammonium emission was up to 1.23 million tons per year [27]. Thus, it was necessary to remove nitrogen from different ammonium wastewater in China. To fulfill the demand, the research groups in China started to carry out ANAMMOX research and obtained significant outcomes. As a part of continuation of ANAMMOX research, nitrogen removal rate higher than 10 kg-N/m^3^/d was obtained by a significant number of researchers in China [26, 28–30]. Till now, the highest NRR in the world was reported by Chinese research group which was 74.3–76.7 kg-N/m^3^/d at hydraulic retention time (HRT) of 0.16 h [28]. Besides nitrogen, sulfur is another necessary nutrient for all living creatures, which means that no one can grow and reproduce without them [31, 32]. Ammonium and sulfate are coexistent in seawater and sediments with the average concentrations of 100 mM and 20 mM, respectively, and this coexistence offers a basic condition for sulfate-dependent anaerobic ammonium oxidation [33]. Using this basic condition, simultaneous removal of ammonium and sulfate was discovered in 2001 in an anaerobic fluidized-bed reactor by Fdz-Polanco et al. [34] and they reported that 80% sulfate was converted to elemental sulfur accompanied by ammonium oxidation to dinitrogen gas. Subsequently, the existence of sulfate-dependent anaerobic ammonium oxidation has been confirmed by other researchers [14, 35–38]. Sulfate-dependent ANAMMOX process was also reported by a number of researchers in China [14, 15, 37–39].

The research groups from the mainland, Taiwan, and Hong Kong of China have conducted a lot of investigations on ANAMMOX process including density and settleability mechanism of sludge, modeling of packing pattern, control mechanism for floating sludge, sequential biocatalyst addition for inhibition recovery, process recovery mechanisms illustration, and so on to enhance the ANAMMOX process performance and stability of the process. After nearly 20 years of hard work, the full-scale application of the ANAMMOX has been successfully implemented for treatment of monosodium glutamate wastewater, pharmaceutical wastewater, and landfill leachate [40–44]. A series of progress in bacteria and process development were also forwarded, which contributed significantly to help us understand the process, and finally instigated researchers to operate and control the novel autotrophic process. The objective of this review is to present a detailed comparative summary of previous and current researches on the progress in ANAMMOX bacteria discovery, process development, and the full-scale application in China.

## 2. Recent Progress of ANAMMOX Bacteria in China

The ANAMMOX process is carried out by a group of ANAMMOX bacteria which are monophyletic group of bacteria under Planctomycetes phylum, Brocadiales order, and *Candidatus* taxonomic component [12]. Till now at least 5 genera and 13 species have been identified using culture-independent molecular techniques [12] including *Candidatus *“Brocadia” (*Ca. *“Brocadia anammoxidans”, *Ca. *“Brocadia fulgida,” and *Ca. *“Brocadia sinica”); *Candidatus *“Kuenenia stuttgartiensis”; *Candidatus *“Scalindua” (*Ca*. “Scalindua brodae”, *Ca*. “Scalindua wagneri”, *Ca. *“Scalindua sorokinii”, *Ca. *“Scalindua arabica”, *Ca. *“Scalindua sinooilfield,” and *Ca*. “Scalindua zhenghei”); *Candidatus *“Anammoxoglobus” (*Ca. *“Anammoxoglobus propionicus” and *Ca. *“Anammoxoglobus sulfate”); *Candidatus *“Jettenia asiatica”.

*Candidatus* Brocadia, Kuenenia, Scalindua, Anammoxoglobus, Jettenia genera have been isolated from different wastewater treatment systems [6, 17, 19, 45–47]; only one genus (*Candidatus* Scalindua) seems to be predominant in marine ecosystems ranging from arctic to tropical regions [48–50]. K. stuttgartiensis is fresh water ANAMMOX bacterium but could adapt to high salinity (up to 3%) [51]. Addition of organic acids like propionate or acetate favored to proliferate the Brocadia fulgida or Anammoxoglobus propionicus species with low fluxes of organic matter [17, 46].

Three new species of ANAMMOX bacteria (*Candidatus* Brocadia sinica, *Candidatus* Anammoxoglobus sulfate, and *Bacillus benzoevorans*) have been discovered in China. Among these three species *Candidatus* Brocadia sinica was isolated from ANAMMOX reactors and other two species (Anammoxoglobus sulfate and *Bacillus benzoevorans*) were identified in sulfate removal reactors.

### 2.1. *Candidatus *Brocadia sinica

Hu et al. [13] operated 8 bioreactors with highly compact granulated sludge in different physicochemical conditions. Although the reactors were operated in distinct variations in temperature (9–27°C) or influent wastewater (inorganic medium, glucose, or glutamate addition), these variations did not influence the inhibition of ANAMMOX bacteria in reactor. Vice-versa, these changing conditions play an important role to grow up large population of the species which only can adapt to this changing situation. Especially, a new ANAMMOX bacterial species was enriched in five reactors among operated eight reactors, which was then provisionally identified as “*Candidatus *Brocadia sinica” according to the taxonomic guidelines. This indicated that ANAMMOX bacteria were found to be adapted to varying seeding influent conditions [51, 52].

### 2.2. Sulfate-Dependent ANAMMOX Bacteria

The research groups in China have discovered two novel species which are inevitable for sulfate removal and conducive for simultaneous ammonium and sulfate removal process. Liu et al. [14] discovered a new species called “Anammoxoglobus sulfate” which was the functional community in reactor to remove ammonium and sulfate concomitantly. Anammoxoglobus sulfate mainly complete the conversion of ammonium and sulfate producing nitrite as intermediate product and then the nitrite diffused from cell to reactor solution across the biological membrane. At the same time, other functional Planctomycetes bacteria performed the traditional ANAMMOX process because both nitrite and ammonium were available in the reactor due to diffusion. Another report showed that Cai et al. [15] conducted the isolation, identification, and characterization of an autotrophic bacterial strain for simultaneous anaerobic ammonium and sulfate removal and identified a new species. The newly discovered species of ANAMMOX bacteria was identified as “*Bacillus benzoevorans*” which was able to use sulfate for anaerobic ammonia oxidation. The identified species will be conducive to the understanding of microbial metabolism of sulfate-dependent ANAMMOX and will enrich microbial resources.

**Figure 1:**
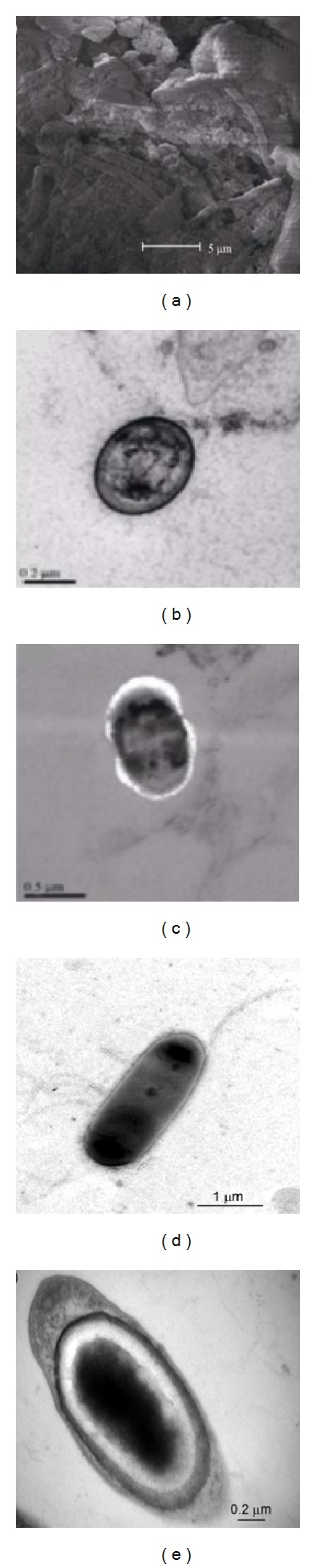
Morphology of sulfate-dependent sludge: (a) SEM, ((b)–(e)) TEM ((a) sludge microbes, (b) Cocci, (c) Bacilli, (d) morphological characteristics of *Bacillus benzoevorans* (×30000), and (e) structural characteristics of *Bacillus benzoevorans* (×70000)) [15, 37].

The sulfate-dependent ANAMMOX bacteria are rod-shaped with flagellum and spore, having a size of (0.7–1.0) × (2.4–3.5) *μ*m. The colony on the plate appeared light yellow, round with a diameter about 1 mm, whose surface was wet and smooth. Scan electron microscopy (SEM) displayed that the sulfate-dependent sludge was dominated by chains of Bacilli and Cocci. The diameter of Cocci was around 0.9 *μ*m and Bacilli diameter was around 0.8 *μ*m, but length varied from 1 to 1.2 *μ*m. Bacterial cells were with inclusions (Figures 1(a)–1(c)) [37]. Another report agreed that sulfate removal *Bacillus benzoevorans* was rod-shaped, spore forming with flagellum which was discovered (Figures 1(d)–1(e)) by Cai et al. [15].

### 2.3. Natural Distribution of ANAMMOX Bacteria in China

ANAMMOX bacteria have been detected in various natural habitats such as anoxic marine sediments and water columns, freshwater sediments and water columns, terrestrial ecosystems, some special ecosystems (e.g., petroleum reservoirs, leachate, pharmaceutical waste, and mangrove), and wastewater treatment systems. ANAMMOX bacteria enumerated, isolated, and identified from different ecosystems in different areas of China have been illustrated in Table 1.

The aforementioned data (Table 1) showed that ANAMMOX bacteria were frequently identified from different ecosystems of different regional areas including terrestrial (soil, wetland) ecosystem, aquatic ecosystem (lake, estuary, river, sea, etc.), and some specialized ecosystem (oil reservoir, MSG wastewater and pharmaceutical waste, landfill leachate, etc.). In most cases, Scalindua and Brocadia were frequently isolated and identified while another genus of ANAMMOX bacteria was identified randomly. So, it is clear that ANAMMOX bacteria were widely distributed in China and, among them, Brocadia was predominant in fresh water and Scalindua in sea water.

## 3. Development of ANAMMOX Process in China

Most of the researchers used synthetic waste waters as influent and other researchers used natural waste (monosodium glutamate waste water, sea, lake, river water, etc.) as influent. Synthetic wastewater was mostly prepared as influent with the addition of ammonium and nitrite in the form of NH_4_Cl/(NH_4_)_2_SO_4_ and NaNO_2_. A variety of reactor types including sequencing batch reactor (SBR) [77], upflow anaerobic sludge blanket (UASB) [78], continuous stirred tank reactor (CSTR) [79], and membrane bioreactor (MBR) [80] have been tested for ANAMMOX startup and all show advantages and disadvantages. Usually 20 days matured sludges are better for the reactor start up. The carmine color or bright red color settled sludge was more active than the floating sludge.

Reactors startup occurred by depositing the seeding sludge in reactor and subsequent addition of natural or synthetic waste waters in reactors. During the reactor startup influent concentrations below 100 mg/L of nitrite, long HRT (few days to few hours), and completely anaerobic and dark conditions are conducive for quick and effective reactor startup. During reactor startup most of the researchers maintained NH_4_–N : NO_2_–N at 1 : 1.1 to 1 : 1.32 and outflow concentration below 30 mg/L. The temperature was set at 35 ± 1°C according to Tsushima et al. [8], and the influent pH was controlled within the range of 6.8–7.0 [81].

### 3.1. High-Rate Nitrogen Removal Performance

ANAMMOX research is promising and booming in China. Many researchers focused on high performance of ANAMMOX activity, some researchers emphasized high loading rate, and some other researchers plunged to discover something new and promising for ANAMMOX development. As a result, different kinds of ANAMMOX activity in lab scale to full scale have been carried out and a lot of findings have been achieved in China. The significant findings and crucial development in ANMMOX research have been highlighted in Table 2.

Tabular data showed that many research groups obtained NRR more than 10 kg-N/m^3^/d which was significantly higher than the reported values all over the world. Chen et al. [29] obtained 57 kg-N/m^3^/d nitrogen removal rate which was significantly a higher value compared to most of the reported values. Tang et al. [28] obtained a super high rate performance with nitrogen removal rate (NRR) of 74.3–76.7 kg-N/m^3^/d at 0.16–0.11 h HRT which was 3 times higher than the previously reported highest value by Tsushima et al. [8]. This was not only top value in China but also in the whole world for laboratory scale reactor performance. Tang et al. [28] also obtained maximum sludge concentration in reactor (42.0–57.7 g-VSS/L) which was 2-3 times higher than previously reported values (0.07 g-VSS/g-NH_4_^+^–N, Trigo et al. [22]; 0.088 g-VSS/g-NH_4_^+^–N, Strous et al. [2] and 0.11 g-VSS/g-NH_4_^+^–N, van Dongen et al. [82]), and the biomass doubling times were also shorter than the value reported in 11 days by Strous et al. [2]. The research reports clarified that the highest performance was directly related to high settling velocity of sludge, high specific ANAMMOX activity (SAA), and high ECP and Heme *c* contents. So, it could be assumed that high reactor performance would be obtained with the following strategies.

- The biomass concentrations in the reactors more than 37 g-VSS/L lead to compact packing of sludge which directly favored the high reactor performance.
- The well-settled granules accumulation in reactor contributes to the super high volumetric nitrogen removal rates even at extremely high NLRs and short HRTs.
- The high value of specific ANAMMOX activity (up to 5.6 kg-N/kg-VSS/d) and short doubling time of bacteria (below 11 days) could be regarded as the key factors to obtain the super high rate performance.
- Relatively high ECP and Heme *c* content and low PN/PS ratios contributed to the ANAMMOX granulation which leads to high performance.

### 3.2. Sulfate-Dependent ANAMMOX Process

Simultaneous removal of sulfate and ammonium by ANAMMOX process is a new biotechnology in wastewater treatment in which ammonium is oxidized with sulfate as electron acceptor under anoxic conditions. The new anaerobic ammonium and sulfate removal process can offer a great future potential for an energy-saving and environment-friendly alternative for simultaneous nitrogen and sulfate removal from wastewater. In 2001, the simultaneous ammonium and sulfate removal process was firstly discovered in an anaerobic fluidized-bed reactor by Fdz-Polanco et al. [34]. According to the report 80% sulfate was converted to elemental sulfur accompanied by ammonium oxidation to dinitrogen gas.

#### 3.2.1. Possible Reaction Mechanisms for Sulfate-Dependent ANAMMOX

Anaerobic ammonium oxidation with sulfate is a microbiological sulfate reducing reaction. The sulfate-dependent isolate used carbonate as carbon source, ammonium as energy and nitrogen source, and sulfate as electron acceptor. Both SO_4_^2−^ and NH_4_^+^ were chemically stable under anaerobic conditions, but the reaction could be possible in the presence of biological catalyst (sludge). The spontaneity of a chemical reaction is associated with its free energy change (Δ*G*) [83]. Free energy for NH_4_^+^ is −79.37 KJ/mol and for SO_4_^2−^ is −744.63 KJ/mol, respectively. Δ*G* ^*θ*^ of the sulfate and ammonium reaction (2NH_4_^+^ + SO_4_^2−^ → N_2_ + S + 4H_2_O (Δ*G* ^*θ*^ = −45.35 KJ/mol)) is −45.35 KJ/mol. So, it indicated that the simultaneous reaction could occur. Practically the spontaneity of chemical processes is conducted by Δ*G* if the absolute value of its Δ*G* ^*θ*^ is less than 46 KJ/mol [20]. Thus, Δ*G* at different substrate concentrations is calculated according to the equation Δ*G* = Δ*G* ^*θ*^ + *RT*ln⁡*Q* (*Q* is reaction quotient). If the Δ*G* value under tested conditions changed from positive to negative by increasing the substrate concentrations, the reactions between NH_4_^+^–N and SO_4_^2−^ were therefore enhanced based on this principle. On the other hand, optimal nutrient and environmental conditions Δ*G* also play an important role in the process realization. Sulfate reducing ANAMMOX bacteria (SRB) are a kind of obligate anaerobic bacteria and only viable when the oxidation reduction potential (ORP) is below −100 mV, which was fulfilled by flushing the reactor with N_2_ [84]. This higher substrate concentration and lower (below −100 mV) oxidation reduction potential (ORP) phenomenon contributed to the simultaneous sulfate and ammonium removal.

Fdz-Polanco et al. [34] explanation showed that autotrophic anaerobic ammonium oxidation and sulfate deoxidation occurred by three consecutive biochemical reactions: (1), (2), and (3).

(i) Ammonium was partly oxidized by sulfate and vice versa sulfate was deoxidized to produce nitrite and sulfide.
(ii) Then part of the produced nitrite was reduced by sulfide and was converted to dinitrogen gas and elemental sulfur.
(iii) Finally, nitrite-dependant anaerobic ammonium oxidation (ANAMMOX) took place.
(iv) Being in a reduced form, sulfide can be oxidized into either elemental sulfur or sulfate, depending on the initial ratio between sulfide and nitrite [85]. The main products are likely to be elemental sulfur and sulfate at a ratio of 3 : 4 (4). Sulfide and nitrite molecular ratio must be critically controlled to prevent sulfate production:
  (1) 3SO42−+4NH4+→3S2−+4NO2−+4H2O+8H+(ammonium  oxidation  process)
  (2) 3S2−+2NO2−+8H+→N2+3S+4H2O  (nitrite  reduction  process)
  (3) 2NO2−+2NH4+→2N2+4H2O(ANAMMOX  process)
  (4)  3S2−+4NO2−+4H2O→SO42−+2S+8OH−+2N2
  (5)  NH4++12SO42−→12N2+12S+2H2O

The experimental data revealed that high substrate concentrations and low oxidation-reduction potential (ORP) enhanced the biological reaction. From the above reactions we can conclude that ammonium reacted with sulfate and was oxidized to nitrite (intermediate product) inside sludge granule (bacterial cell) by oxidation process and concomitantly sulfate is deoxidized. Then, the produced nitrite diffused from sludge (bacterial cell) to reactor and reacted with ammonium in the reactor by ANAMMOX process and eventually produced nitrogen gas. In the terminal stage ammonium and sulfate reacted with each other and were converted to nitrogen gas and solid sulfur.

#### 3.2.2. Sulfate-Dependent ANAMMOX Researches in China

Most of the research groups had used (NH_4_)_2_SO_4_ and NaNO_2_ substrates as influent in reactor for N and S source, respectively [15, 37, 38]. Vice versa some researchers used only (NH_4_)_2_SO_4_ as sole substrate in influent for N and S source [14].

Sulfate-dependent ANAMMOX bacterial growth is too slow or the doubling time is too long, and thus it takes more time to start up the reactor. The anaerobic digested sludge was cultivated in an anaerobic reactor for three years, and then the sludge became capable to oxidize ammonium with sulfate anaerobically. The sludge volume increase of sulfate reducing ANAMMOX was trivial because a little bit biomass development was achieved after a long-term reactor operation. Reaction between sulfate and ammonium that occurred rarely was difficult to observe, but it became possible only with the coexisting of sulfate reducing and ANAMMOX bacteria. Although some research groups revealed the reaction mechanisms for simultaneous removal of ammonium and sulfate, only very few researches on this topic were carried out due to requirement of long-term operation and minuscule success probability. It is found that some research groups conducted research on this and disclosed promising idea and application. The reports of recent researches have been presented in Table 3.

Cai et al. [15] investigated simultaneous ammonium and sulfur removal ANAMMOX process and phylogenetically analyzed a sulfate reducing strain which was related to *Bacillus benzoevorans*. The investigation also revealed that the maximum 44.4% and 40% ammonia and sulfate removal rates even at pH 8.5 and stoichiometric ratio 2.01 : 1 developed by Fdz-Polanco et al. [34] were obtained, respectively. Yang et al. [38] also reported 40% and 30% removal efficiencies of ammonium and sulfate at molar ratio of ammonium to sulfate consumption of 2 : 1, respectively, in an anaerobic bioreactor filled with granular activated carbon. Another report of Liu et al. [14] disclosed that a new species of Brocadia genus, namely, “Anammoxoglobus sulfate” which was discovered from a ammonium and sulfate enriched niche and revealed that this species played a critical role in the ammonium oxidization and sulfate reduction. The species mainly completed the conversion of ammonium and sulfate and then produced an intermediate product nitrite which diffused from bacteria (sludge) to reactor solution across the biological membrane. Then some other functional Planctomycetes bacteria perform the traditional ANAMMOX process due to presence of ammonium and nitrite. So, the investigation indicated that the full conversion of ammonium and sulfate occurred with the coexistence of ANAMMOX and sulfate reducing bacteria. In this investigation nonwoven rotating biological contactor reactor was preferred over some other types of reactors because it combines the advantages of a biofilm reactor (large specific surface area) and adsorption characteristics with the improvement of the gas-liquid transfer rate (good mixture of substrates and flux). Zhang et al. [37] cultivated the anaerobic digested sludge in an anaerobic reactor for three years and found that after long-term operation the sludge became capable to oxidize ammonium with sulfate anaerobically. The obtained results significantly contributed to the sulfate-dependent ANAMMOX research in the world.

Sulfate reducing ANAMMOX process was sensitive to pH and temperature, and it is reported that reactor performance was inhibited at pH and temperature higher than 8.5 and 30°C [15], respectively. Liu et al. [14] reported that simultaneous removal process might be hindered if there is no coexistence of sulfate reducing bacteria and autotrophic ANAMMOX bacteria. According to Yang et al. [38] ammonium and sulfate removal efficiencies might be affected by some middle medium, such as nitrite, H_2_S, and sulfur. Supporting to this report, Fdz-Polanco et al. [34] reported a complete loss of ANAMMOX activity when the nitrite concentration remained above 5 mM (70 g NO_2_^−^–N/m^3^) for a long period (12 h). Another report surmised that sulfide might toxic to microorganisms [86].

#### 3.2.3. Future Prospect of Sulfate-Dependent ANAMMOX Process in China

It can be proposed that sulfate-dependent ANAMMOX researches have an inspiring future and development and application in China with the following prospects.

- Comparing to ANAMMOX bacteria, the biomass production or doubling time of sulfate-dependent ANAMMOX is too slow. This limitation could be overcome by enrichment of reactor with special care.
- Newly discovered two species, *Bacillus benzoevorans* and Brocadia “Anammoxoglobus sulfate,” could be used ubiquitous for simultaneous removal of sulfate and ammonium.
- Pure chemical reaction between ammonium and sulfate without microorganisms was not possible. So, massive biomass production could open a new way for vigorous application.
- Sulfate-dependent ANAMMOX performed good in coexistence condition of biomass which could play an important role in extensive reactor performance improvement.

### 3.3. ANAMMOX Process with Sequential Biocatalysts Addition

Tang et al. [40] observed that the pharmaceutical wastewater possessed severe acute toxicity with a relative luminosity value of 3.46 ± 0.45% and the conventional ANAMMOX process was not suitable for nitrogen removal from this wastewater due to cumulative toxicity. Finally, the toxicity of the pharmaceutical wastewater was successfully avoided with the introduction of a novel SBA-ANAMMOX process (ANAMMOX process with sequential biocatalyst addition) and the nitrogen removal rate was also significantly enhanced to 9.4 kg-N/m^3^/d (by adding 0.025 g VSS/d fresh seeding sludge in reactor) [40]. So, the application of SBA-ANAMMOX process in refractory ammonium-rich wastewater is encouraging.

There may be a question of how the sequential addition of fresh sludge improves the reactor performance. Exogenous addition of high-activity ANAMMOX biomass into the reactor system can supplement the required bacterial amount and also can increase the density of sludge in reactor which eventually enhanced the nitrogen removal performance. Deliberate addition of high-activity ANAMMOX seeding sludge into reactor led to the addition of some unknown growth factors contained in the granules which showed stronger resistance to toxic substances and eventually enhanced the growth of ANAMMOX bacteria [40]. The granules addition subsequently contributed to the high-rate nitrogen removal performance at the low biocatalyst addition rate. So, the answer is that addition of fresh sludge in the reactor increased the settleability, density, and growth conditions of the existing sludge which help to rejuvenate the existing sludge activity and thus improve the reactor performance.

Li et al. [27] disclosed that the returning of washout sludge to the reactor played an important role in prompt recovery of ANAMMOX performance. Yang et al. [87] proposed that addition of fresh ANAMMOX sludge to reactor may improve the living conditions of ANAMMOX bacteria, and, by adding 20.1 g/L (VSS) sludge (on day 135) and 21.0 g/L (VSS) sludge (on day 170) in the inhibited reactor, they obtained increasing nitrogen removal rate from 0.025 to 0.069 kg-N/m^3^/d (on day 135) and from 0.069 to 0.323 kg-N/m^3^/d (on day 170), respectively. By operating the conventional ANAMMOX reactor and SBA-ANAMMOX reactor in parallel, Tang et al. [40] found that the ammonium and nitrite removal efficiencies for SBA-ANAMMOX reactor were 80.4–86.2% and 92.6–94.8%, respectively, while the removal efficiencies for conventional reactor were only 1.9–13.8% and 14.5–34.2%, respectively. This indicated that SBA-ANAMMOX process could successfully overcome the toxic effects of refractory ammonium-rich pharmaceutical wastewater. From different research reports, it could be concluded that sequential sludge addition is an effective and sustainable way to improve the reactor performance. Thus, the cost-effective and high-rate SBA-ANAMMOX process is a promising biotechnology with enormous advantages that could treat ammonium-rich wastewaters effectively.

## 4. Morphological Study of ANAMMOX Granules

### 4.1. General Characteristics of ANAMMOX Granules

**Figure 2:**
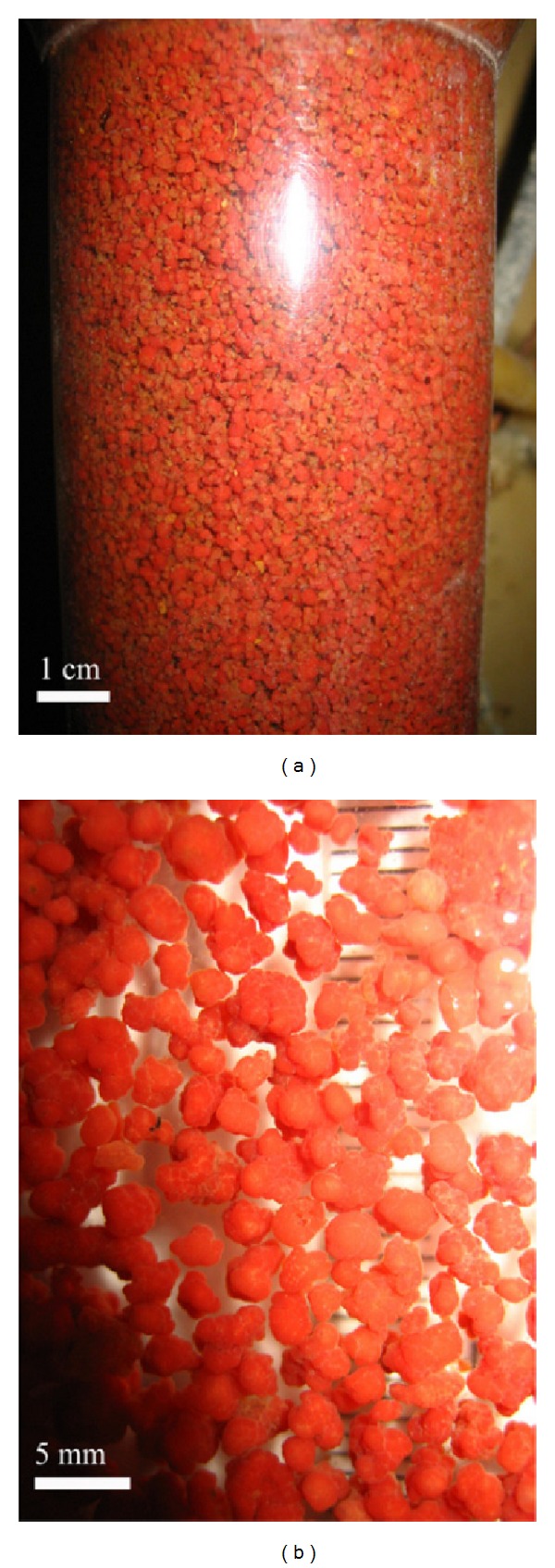
The granules in high-rate ANAMMOX UASB reactor (a) and the image of ANAMMOX granules (b) [88].

*Color*. The color of high performing fresh seeding ANAMMOX sludge tended to be bright red or carmine (Figure 2). Sludge color is an indicator of sludge viability and performance. If the sludge color changed from carmine to pale red or black, it indicated that the sludge activity reduced.

After a long-term reactor operation the sludge became pale red due to a decrease of Heme *c* and became black because the sludge did not get a substrate for a long time.

*Size*. The average diameter of ANAMMOX sludge granule varied from 2.2 to 2.5 mm with VSS/TSS at 87% and among them the larger than 2 mm granules were predominant (68–71%) [28]. Sludge size is also an indicator of reactor performance. Large sludge has low settleability and small sludge has high settleability. High settleability is a good indicator for effective reactor performance. The diameter of the floating granules was 2.31–6.96 mm with an average of 4.58 ± 1.22 mm, while that of the settling granules was 0.86–6.98 mm with an average of 2.96 ± 0.99 mm [89]. Large sludge has more gas pockets and gas volume than small sludge [89]. It implied that the ANAMMOX granules with a diameter larger than 4 mm had a strong trend to float in high rate reactors due to accumulation of dinitrogen gas in gas pockets [28]. At high removal rate more gas produced inside the sludge which lead to decrease the settleability of sludge, and thus larger sludge became floating and smaller sludge remained settled [89]. Floating behaviors of sludge decrease the reactor performance.

*Density*. Sludge density is another indicator of high activity reactor performance. The density of ANAMMOX granules in reactors was measured by Tang et al. [28], and it was about 1.03 g/mL; the specific density of the ANAMMOX granules (91 to 120 g-VSS/L-granules) was also comparable to aerobic granules (40 to 70 g-VSS/L-granules) and high-loaded denitrifying granules (128 to 136 g-VSS/L-granules, Franco et al. [90]). Sludge density is proportional to reactor performance, and alternatively sludge size is disproportional to sludge density.

*Settling Velocity of ANAMMOX Granules*. According to the well-known Stokes equation, the settling velocity of ANAMMOX granules is closely related to their size and density. The increasing size and density might result in a significant increase of settling velocity of granules [91]. The floating ANAMMOX granules did not settle, so only the settling velocity of settling ANAMMOX granules was determined. The settling velocity increased with the increase of sludge diameter and high settling velocity (73 to 88 m/h) was obtained by Lu et al. [92].

*Relationship of Settleability with Structure and Density*. The diameter and the density were two key factors for the settleability of ANAMMOX granules in the reactors. The settling velocity of ANAMMOX granules increased with the increasing diameter, while the density of ANAMMOX granules decreased with the increasing diameter. Mature ANAMMOX granules settled very well at velocities of 41 to 79 m/h, which were similar to the settling velocities of the methanogenic granules (e.g., 52.9 m/h) and at least two times greater than those of the flocculated ANAMMOX sludge [41]. The significant increase in settling velocities indicated that the ANAMMOX granules had a highly dense and compact structure which is more effective for sludge-effluent separation in the treatment system.

### 4.2. Morphology of ANAMMOX Granules

**Figure 3:**
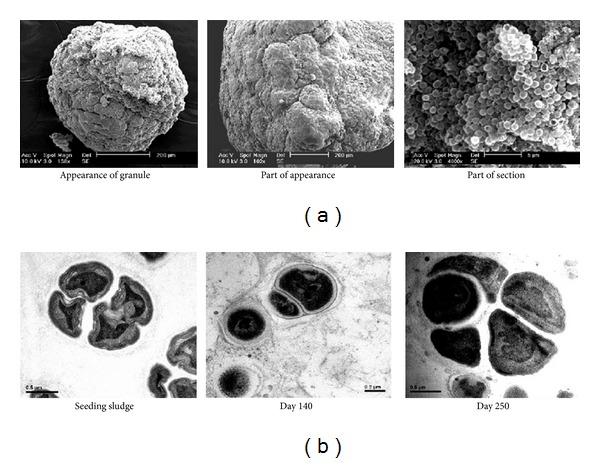
(a) SEM images of the ANAMMOX granular sludge. (b) TEM images of the ANAMMOX bacteria in sludge in the granulation process [41].

The structure of the microbial granules developed in reactors was observed by means of SEM and TEM. The scanning electron micrographs (SEM) showed typical coccoid-shaped cells as the dominant microorganisms on the granule surface embedded in an extracellular polysaccharide (EPS) matrix (Figure 3(a)), very similar to those reported by others [53, 93, 94].  Figure 3(b) showed the typical crescent-shaped ANAMMOX cells obtained via transmission electron micrograph of chemically fixed ANAMMOX cells [95]. The scanning electron micrographs represented by Tang et al. [28] showed that a red-colored mature ANAMMOX granule was characterized by a cauliflower-like shape. The granular surface mainly consisted of spherical and elliptical bacteria; few or even no bacilli and filamentous bacteria were observed in the two reactor enrichments, suggesting that the ANAMMOX bacteria dominated after enrichment [28].

### 4.3. Chemical Properties of ANAMMOX Granules

#### 4.3.1. Extracellular Polymers

Before discovery of granulation mechanism, researchers used single ANAMMOX bacterial cells for reactor operation. After discovery of sludge granulation process, plenty of researchers operated reactors with sludge and pointed out that sludge mediated reactor performances were obviously higher than bacterial mediated reactor performance. So, it was of a great demand to the researchers to explore the possibility of granulation of ANAMMOX biomass and its application for high performance. The EPS containing bacteria played vital role in the formation of granules in bioreactors [96–99]. ECPs could physically bridge neighboring cells by altering the negative charges on bacterial surface [99], and thus granulation may be facilitated by large secretion of ECP. EPSs are distributed throughout the ANAMMOX bacterial surface which attracted scientists to study it with a view of obtaining an efficient, sustainable, and stable sludge granulation for high rate reactor operation.

**Figure 4:**
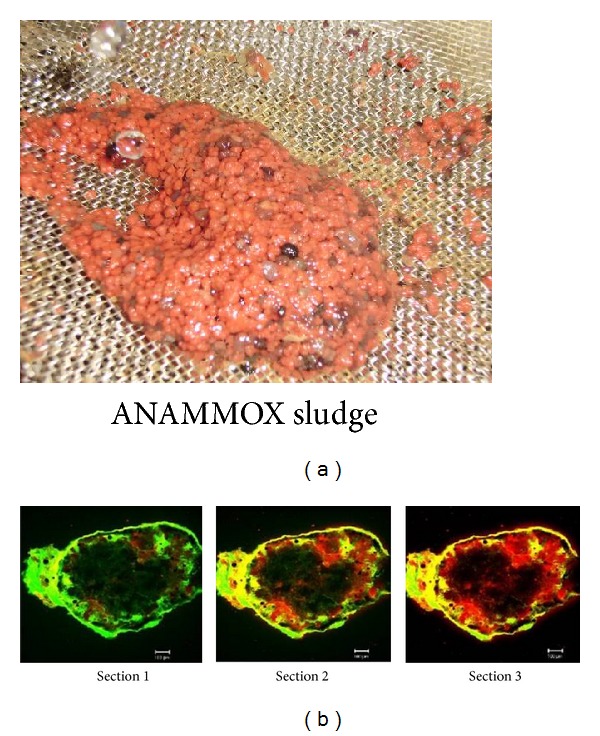
(a) EPSs at outer surface of carmine red ANAMMOX sludge, (b) CLSM images of the 50 *μ*m cryosections of the ANAMMOX granule from surface to center. Cells were stained with SYTO9 (green) and polysaccharides were stained with concanavalin A (red) [28, 41].

The microbial ECPs are a rich matrix of polymers which mainly constituted of polysaccharides and proteins [98]. The EPS of each gram of ANAMMOX granule (in VSS) contained 83.2 ± 7.9 mg carbohydrates and 42.7 ± 6.5 mg proteins granules [41]. The EPS content of autotrophic ANAMMOX granules (125 mg EPS/g-VSS) is significantly higher than EPS content (10–91 mg EPS/g-VSS) of heterotrophic methanogenic granules [41]. The protein/carbohydrate ratio (0.51) of autotrophic ANAMMOX granule is lower than heterotrophic methanogenic granules (1.2–4.0) [41]. This indicated that protein might be the key constituent for methanogenic granules and contrarily carbohydrate might be the major constituent for ANAMMOX granules. This information postulated that carbohydrate might play more important role than protein in the formation of ANAMMOX granules. The large amount of glycosylation proteins encoded by the “*Candidatus* Kuenenia” genome is supportive of this suggestion [17]. Tang et al. [28] determined the ECP content at different nitrogen removal levels and summarized that both polysaccharide and protein contents increased with the increasing nitrogen removal rate (NRR). The investigation also revealed that the polysaccharide contents (71.8 ± 2.3 mg/g-VSS) increased slowly as compared to the protein contents (164.4 ± 9.3 mg/g-VSS). The CLSM analysis (Figure 4) showed mixed patterns of cells and EPS distributions in the ANAMMOX granules [41]. The EPSs were distributed throughout the granules, while the bacteria were mainly situated in the outer layer of the granule.

The proteins to polysaccharides ratio (PN/PS) was generally used to assess the granular settleability and strength [90, 100–104]. Various researchers investigated EPS and pointed out that the higher PN/PS ratio of microbial granules led to lower strength and weaker settleability [100–103]; thus the sludge floating or foaming would occurr easily [90, 104]. The PN/PS ratio of the ANAMMOX granules (0.51) was also low in contrast to other microbial granules (1.2–4.0), recommending a greater granular stability [90]. Tang et al. [28] determined the ECP contents of the floated granules and found that extracellular proteins and PN/PS ratios of the floated ANAMMOX granules (3.88–4.09) at high NRRs and HLRs were significantly higher than the counterparts of well-settled (2.40–2.65) granules. The overproduction of extracellular proteins might be a potential cause resulting in the severe sludge washout from the UASB reactors.

#### 4.3.2. Heme *c* Content

Sludge color is an indicator of high activity ANAMMOX granule, and the sludge color varied from carmine red to pale red, brownish, or black. High-load ANAMMOX granules are uniquely carmine (Figure 4) in color. It was well accepted that Heme *c* played a vital role to develop carmine color in sludge granule. Sludge color was directly related to reactor performance, and thus researchers penetrated their concentration to reveal the role of Heme *c* and its evaluation.

ANAMMOX bacteria have some vital enzymes such as hydrazine synthase (HZS), hydroxylamine oxidoreductase (HAO), and hydrazine oxidase [105] which are rich in ANAMMOX bacteria cell and involved in substance and energy metabolism. Recent researches report postulated that Heme *c* is the indispensible part of these key enzymes of ANAMMOX bacteria [106, 107] and the content of Heme *c* might be involved in ANAMMOX activity and other relative activities [108]. Heme *c* also plays an important role for the development of carmine color of ANAMMOX sludge. It is assumed that the content of heme *c* might be changed with the fluctuation of nitrogen removal rates (NRR), and Tang et al. [28] disclosed that the content of Heme *c* increased significantly with the increasing NRR. Tang et al. [28] showed that the Heme *c* content was 0.7–1.4 mmol/g-VSS at NRR lower than 10 kg-N/m^3^/d and the content dramatically reached 9.7–10.8 mmol/g VSS at NRR higher than 70 kg-N/m^3^/d. Thus the increase of Heme *c* content was associated with the increase of ANAMMOX bacterial numbers, resulting in high specific ANAMMOX activity (SAA). Finally it could be concluded that the changes of Heme *c* content could represent the growth status of ANAMMOX consortia and Heme *c* level could be used to evaluate the relative activity of ANAMMOX microorganisms and to imply the recovery of ANAMMOX reactor performance. Carmine red, grey, and black color sludges are presented in Figure 4(a). The red colored is due to high Heme *c* content, grey color due to low content, and back is due to absence of Heme *c* that occurred by blockage of sludge.

### 4.4. Structure of ANAMMOX Granules and Granulation of ANAMMOX Biomass

#### 4.4.1. Structure of ANAMMOX Granules

**Figure 5:**
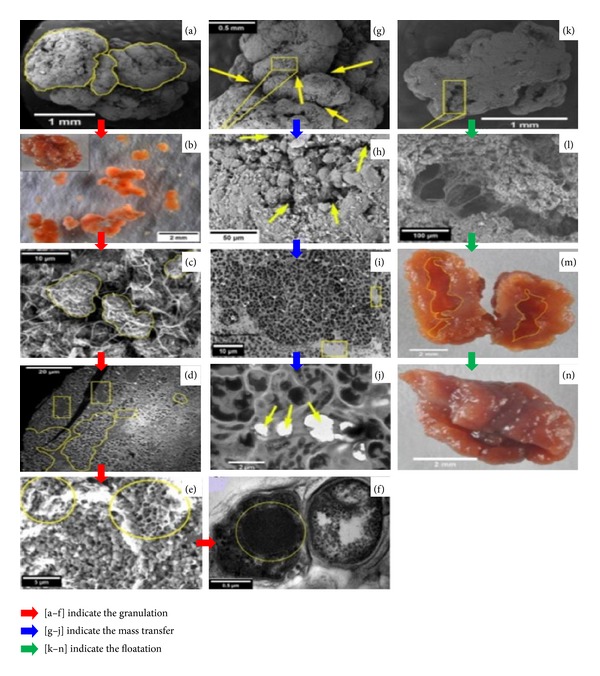
Structures of ANAMMOX granules DMP ((b), (m)-(n)), SEM ((a), (c), (e)–(h), (k), and (l)), and TEM ((d), (f), and (i)-(j)). (a) One ANAMMOX granule composed of subunits (marked with closed curve) and ditches (indicated with arrows) separating the subunits. (b) An ANAMMOX granule (on the top left corner) broken into several subunits. (c) Microbial cell clusters (marked with closed curve) connected by the filamentous bacteria and (d) separated by filamentous bacteria-EPS bands (indicated with rectangles). (e) Interstitial space: the honeycomb-like structures (indicated with circles). (f) ANAMMOX bacteria-like cells (marked with a circle). (g) Ditches (indicated with yellow arrows) between subunits in the exterior of granules. (h) Interstitial voids (indicated with yellow arrows) between microbial cell clusters in the exterior of granules and (i) in the interior of granules (indicated with rectangles). (j) Gas tunnels between microbial cells (indicated with arrows). (k) Small gas pocket (marked with rectangle). (l) Gas pocket of a settling granule formed by inflation of dinitrogen gas. (m) Gas pocket of a floating granule (indicated with closed curve). (n) A broken floating ANAMMOX granule formed by the burst of dinitrogen gas [92].

According to the observation of Lu et al. [92] under light microscope and electron microscope, the structure of ANAMMOX granules included granule, subunit, microbial cell cluster, and single cell (Figure 5).

*(i) Granule*. Anammox granules were irregular in shape with a diameter of 2.96 ± 0.99 mm [92] and the surface of granule was rough. The granule was broken along the furrow and the broken parts were known as subunits. Subunits were linked to each other by EPS and filamentous bacteria.

*(ii) Subunit*. SEM and TEM micrographs of different researches mentioned that filamentous bacteria were predominant both in inner and outer surface of ANMMOX subunits. Subunit or “EPS-filamentous bacteria” bands were segmented into several compartments which were called cell clusters.

*(iii) Microbial Cell Cluster*. In cell clusters microbial cells were aggregated close to each other by EPSs and filamentous bacteria, and the cluster size varied from few to 200 *μ*m. The microbial cell clusters were composed of ANAMMOX bacteria-like cells.

*(iv) Interstitial Voids*. Subunits, microbial cell clusters, or microbial cells were separated from each other by interstitial space which was known as interstitial voids. Interstitial voids were surrounded by EPSs and were filled with water or dinitrogen gas. The sizes of interstitial voids were proportional to granular size and voids could serve as water channel and gas tunnel. In the beginning interstitial voids were filled with water and worked as water channel to transport substrates (NH_4_^+^ and NO_2_^−^) and products and they were 80 nm in diameter [109, 110]. With the activities of microbial cells the transported substrates were converted to dinitrogen gas and this gas accumulated inside the ANAMMOX granules, and the interstitial voids were finally used as gas tunnels for release of dinitrogen gas.

#### 4.4.2. Granulation of ANAMMOX Biomass

In the beginning of ANAMMOX process discovery the low influent loading rate or moderate influent loading reactors were operated with single ANAMMOX bacteria and there were no problem in reactor operation. But when high loading rate reactors operation started with an extremely high upflow velocity, large influent loading rate and large gas production of the ANAMMOX biomass were washed out with effluent due to poor settleability [111]. So, granulation of ANAMMOX biomass was inevitable to overcome the biomass washout and to enhance reactor performance. Thus researchers emphasized this and their microscopic observations assumed that granulation process accomplished with the sequential aggregation of microbial cells, cell clusters, and subunit.

**Figure 6:**
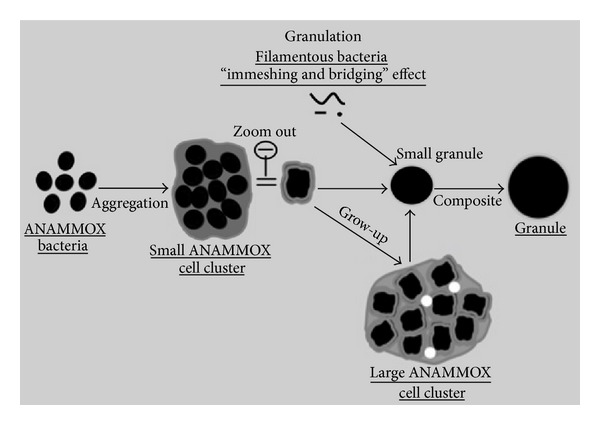
The hypothesized mechanism for granulation and floatation of ANAMMOX biomass. Underlined are the microbial structures observed in this study [92].

ANAMMOX sludge granulation started from ANAMMOX bacterial cell. With an extremely slow growth rate the ANAMMOX bacteria have an affinity to accumulate EPSs [28] and this leads to granulation [22, 112]. ANAMMOX granule has been investigated thoroughly in various bioreactors [22, 82, 113–116], but there is very few studies and findings about the granulation mechanism. According to microscopic observation of Lu et al. [92] the granulation of ANAMMOX biomass granulation (Figure 6) in high-rate reactors could be accomplished in three consecutive steps.

*(i) Formation of Microbial Cell Cluster*. In a high influent loading reactor the bacteria collide with each other due to high flow rate and high shear force. At the time of collision, the bacteria are entrapped with each other by their immobilized sticky EPSs and form an aggregate of bacterial cell called cell cluster.

*(ii) Formation of ANAMMOX Subunit*. Then the microbial cell clusters were assembled together with the help of filamentous bacteria to form an aggregate of cell cluster which is called ANAMMOX subunits. The filamentous bacteria departed from ANAMMOX subunits, and then gas tunnel developed in the interstitial space among microbial cell clusters.

*(iii) Formation of ANAMMOX Granule*. The ANAMMOX subunits in the reactors collided with each other due to the high inflow rate and high gas production and then assembled together with immobilized EPSs and finally produced ANMMOX granules (Figure 6).

### 4.5. Floatation and Control of ANAMMOX Granule

#### 4.5.1. Floatation of ANAMMOX Biomass

All of the ANAMMOX granules have gas tunnels and gas pockets. In high loading reactors with high activity of ANAMMOX bacteria, the gas bubbles are produced in gas tunnels and gas pockets. The gas bubbles entrapped in gas pockets were supposed to be the key factor to cause sludge floatation. After the hollows were filled with gas bubbles, the ANAMMOX granules would float and be washed out of reactor, leading to failure of ANAMMOX process. The granule floatation was found to be an important factor that causes instability or even collapse of ANAMMOX reactor. Facing these problems some Chinese research groups [92, 113] conducted research and revealed the effective strategy to overcome the floatation and to restore reactor performance.

**Figure 7:**
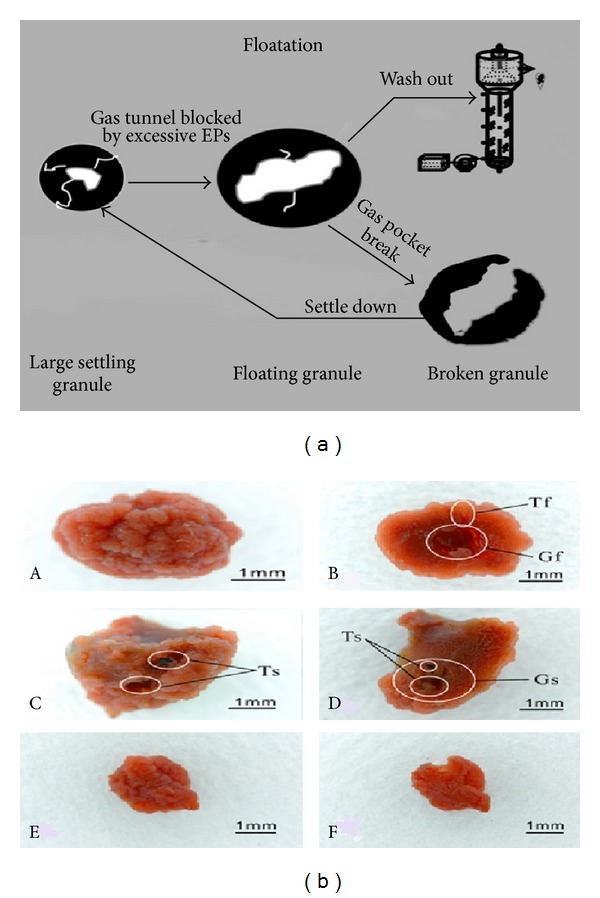
(a) The hypothesized mechanisms for floatation of ANAMMOX sludge, (b) granules in different operation phases: floating granules (A, B), settling granules (C, D), and mechanically broken granules (E, F) in reactor. Both the floating and settling granules contained gas pockets marked G_f_ and G_s_ and gas tunnels marked T_f_ and T_s_, respectively [92, 113].

Inside the floating granules the gas tunnels were closed and inside settled sludge the tunnels were opened. This phenomenon suggested that the gas inside settled sludge could be released easily but the gas inside floating sludge could not be released due to clogging of the gas tunnels. As a result the sludges were floated at high dinitrogen releasing stage and these floatings declined the reactor performances. So, there was a necessity to find out the reason of sludge floatation and control mechanism of floatation for restoration of reactor performance. The floatation of ANAMMOX biofilm was extensively investigated and mechanism of sludge floatation (Figure 7) could be explained in three sequential phases [92] such as (i) formation of gas tunnel, (ii) formation of gas pocket and (iii) floatation of sludge granule.

*(i) Formation of Gas Tunnel*. It has been reported that the ANAMMOX bacteria tend to grow together [22] and thus the ANAMMOX granules were supposed to develop from small sludge granules by adhering to each other, and hollow space inside granules was supposed to develop from the gaps between small sludge granules. The substrates were (ammonia and nitrite) diffused into the bacterial cells from reactor, and subsequently dinitrogen gas was produced inside the sludge due to bacteria activities and accumulated as gas bubbles. Then the gas bubbles tend to be released from sludge through the interstitial gaps between small sludge granules and thus create gas tunnels. Microscopic observation reported that a lot of gas tunnels developed for releasing gas outside of the sludge through these tunnels. The gas tunnels inside settled sludge were open, but inside floating sludge were closed.

*(ii) Formation of Gas Pocket*. With increasing influent rate and high reactor performance, extensive dinitrogen gas was produced. The produced dinitrogen gas entrapped with ANAMMOX granules for maintaining a balance of production and release [114]. The excessive dinitrogen gas inside the granules moved to and fro which created a larger resistance and higher pressure. These larger resistance and higher pressure were forced to enlarge the loose of interstitial voids between cell clusters of subunit and thus led to develop gas pocket. The gas pockets inside the granules were connected to outside environment through gas tunnels.

*(iii) Floatation of ANAMMOX Granules*. According to research reports EPSs production increased with the increasing loading rates [28] and thus extensive EPSs were produced at high loading rates which led to clog the gas tunnels. High loading rate favored the ANAMMOX sludge to produce huge amount of dinitrogen gas which could not be released outside of sludge through the tunnels due to clogging of gas tunnels [113]. As a result, the excessive gas bubbles were entrapped inside gas tunnels. The entrapped dinitrogen gas surpassed a critical value (5.7%, V/V) and thus the density of granules would be lower than that of water, which led to the floatation of ANAMMOX granules.

#### 4.5.2. Control Strategy for Granule Floatation

Chen et al. [113] assumed that the floating granules have blocked gas tunnels and gas pockets and thus the dinitrogen gas could not be released from sludge which caused floating of the sludges. Then the floating sludges were washed out with effluent from reactor which caused deterioration of reactor performance and thus lead to failure of ANAMMOX process. This washout obstacle compelled the researchers to find out the possible way to overcome this obstacle.

The floating granules were taken out of the reactor and broken into small pieces and returned into reactor to overcome the frequent floating problem of ANAMMOX sludge. After the reactor was operated with the control strategy, the floatation and washout of ANAMMOX sludge became constrained and the performance improved. The broken granules (BGs) had various size with diameters mainly of 0.5–2.6 mm and the wet density of broken granules was higher than that of settled granules, reaching 1047.7 ± 5.1 mg/L [113]. The control strategy improved the performance of ANAMMOX reactor. So, “collecting-breaking-returning” procedure (Figure 7) was suggested to be an effective control strategy to solve granule floatation and improve reactor performance. Alternatively the collecting-breaking-returning procedure in some cases might disturb the microbial ecology (death of bacteria, reduction of ANAMMOX activity, loss of biomass particles, etc.) inside granules and impair the ANAMMOX activity. Furthermore, the recovery of the reactor performance might be a bit long since the reformation of ANAMMOX granules was needed.

### 4.6. Packing Pattern and Packing Density of ANAMMOX Sludge

The pore volume and sludge concentration inside ANAMMOX reactors are related to the packing patterns of ANAMMOX granules. The packing patterns are characterized with the coordination number, and packing pattern varied depending on coordination numbers. Previous research reports explained that packing patterns were responsible for pore volume and sludge density, and subsequently sludge density directly led to reactor performance. According to the reports the sludge density 52–55% [88] showed the maximum reactor performance. Different types of packing patterns represent different sludge density and variable reactor performance. So, there was a need to find out the appropriate packing pattern for optimum reactor performance.

Tang et al. [88] initially developed the packing pattern of granular sludge following the principles and theories of crystal lattice patterns [118]. A series of relationships among packing density, sludge concentration, coordination number, nitrogen removal performance, and HRT were evaluated. Finally, the mathematic equation of the packing pattern concerning the nitrogen removal rate (*R*), sludge concentration (*C*_*X*_), and the substrate loading (*c*) was constructed, as shown in (6). Depending on this packing pattern, the nitrogen removal performance could be illustrated by the sludge concentration and substrate load:

(6) $$\begin{array}{c} R=0.527C_{X}q{\left(1-\frac{C_{X}}{\rho \;\text{granule}}\right)}^{25.0}{\left(\frac{C_{X}}{\rho \;\text{granule}\cdot \text{SV}}\right)}^{0.16}C^{0.23}, \end{array}$$

where *q* is specific activity of the sludge, *ρ* granule is the density of granular sludge, and SV is the sludge volume.

The reactor performance could be evaluated with the developed packing pattern. Sludge density, pore volume, and sludge concentration in reactor could be investigated with the help of packing pattern. Based on the sensitivity analysis, the following conclusions could be drawn. When *C*_*X*_ is equal to 37.8 g/L, the sludge concentration and substrate loading rate were equally important in contributing to the high conversion capacity of the ANAMMOX UASB reactor. When *C*_*X*_ was less than 37.8 g/L, *C*_*X*_ was more sensitive than *c*, indicating that it was more effective to enhance sludge concentration for improvement of conversion capacity while, when *C*_*X*_ was higher than 37.8 g/L, *c* was more sensitive, which meant that elevating substrate loading was an advisable strategy for ANAMMOX reactor operation.

## 5. Application of ANAMMOX Process in China

**Figure 8:**
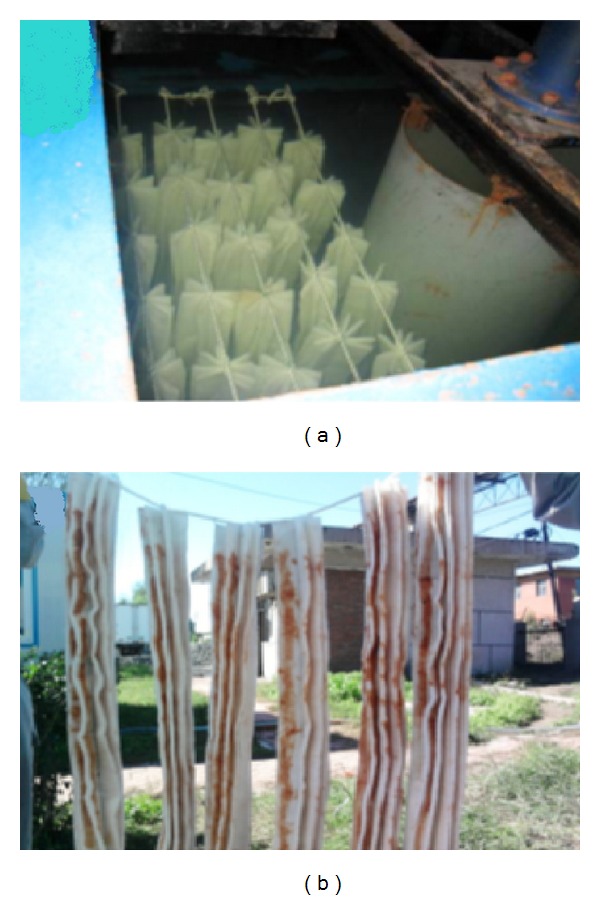
Images of the pilot-scale ANAMMOX reactor (a) and packing materials (b) [117].

In China, ANAMMOX research area is growing day by day. Most of the researches have been conducted in laboratory scale. Some ANAMMOX research groups also carried out ANAMMOX research in pilot-scales and found significant outcomes. Different research groups are operating pilot-scale ANAMMOX process to treat different types of waste and finally succeeded to protect the ecosystem with their maximum capability. The pilot-scale operation is at the point from where the researchers opted to devise for full scale operation. Some pilot-scale ANAMMOX applications have been highlighted in Table 4 and Figure 8.

The above-mentioned data (Table 4) showed that pilot scale reactors were operated with working volume that ranged between 0.020 and 22.5 m^3^, and different types of waste water were treated by applying this process. This implied that pilot-scale reactor operation in China is significant in the transitional point from the development point of view. An et al. [117] successfully operated a pilot-scale ANAMMOX reactor to treat dry-spun acrylic fiber wastewater (DAFW) which is depicted in Figure 8. By controlling the reactor temperature and SS in influent, the removal efficiencies of ammonium and nitrite were 85% and 90%, respectively.

Nitrogen waste is one of the vital pollution problems in China. With the increase of Chinese economy the ANMMOX researches tend to move in full-scale treatment plant. So, China is the promising and largest market for ANAMMOX waste treatments. In the Netherlands, the first full-scale granular ANAMMOX reactor was commenced at the wastewater treatment plant of Water board Holland se Delta in Rotterdam in 2006 [16, 99]. This full-scale ANMMOX treatment plant was the milestone for commercial application of ANAMMOX process. The first full-scale reactor volume was 70 m^3^ which was 7000-fold from 10 L lab-scale experiment. So far, more than 30 full-scale divergent plants are in operation throughout the world, mostly in Austria, China, Japan, the Netherlands, and USA. All of these plants were established emphasizing ANAMMOX process and finally has become a commercial technique. In 2009 Environmental Technology (Shanghai) released the news that an agreement had been reached to build the world's largest ANAMMOX based wastewater treatment plant in China [44].

**Figure 9:**
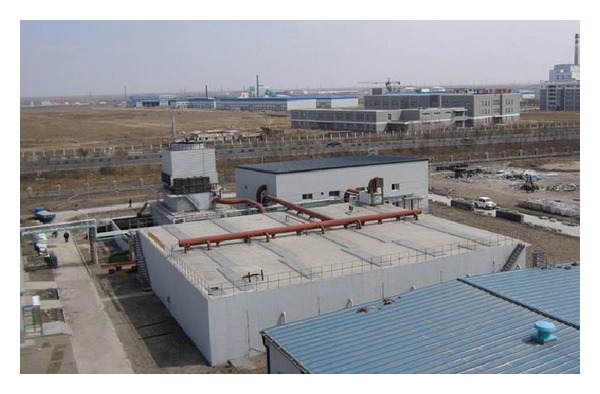
Full-scale 11,000 kg-N/d one-step ANAMMOX installation at the Tongliao Meihua Company in China, Courtesy Paques [121].

At the Tongliao Meihua industrial complex, one step ANAMMOX reactor with design capacity of 11,000 kg-N/d has been implemented by Paque which could treat the effluent from monosodium glutamate production (Figure 9) [121]. This is one of the largest treatment plants and is ten times larger than the largest plant built before in 2008 in China. Another 11 ANAMMOX plants were implemented by Paques, seven of which are located in China (Table 5). As the world's biggest developing market, China contributes significantly towards commercialization of ANAMMOX process.

Besides imported technology, some research groups in China were set to operate with full scale ANAMMOX process with their own possessed sludge, expertise, and technology. It came to know that Zhejiang University ANAMMOX research group have succeeded in implementing two full scale ANAMMOX treatment plants for treatment of monosodium glutamate wastewater (60 m^3^) in Yiwu City and pharmaceutical wastewater (10 m^3^) in Dongyang City, Zhejiang Province, China. Some other research groups also opted to set up full scale application. These initiatives are the clear indication that China already have achieved dramatic progress in ANAMMOX research and very near to be one of the leading group in ANAMMOX technology.

## 6. Conclusion

ANAMMOX process was considered to be one of the most sustainable pathways for nitrogen removal from ammonium-rich wastewater. During the past decades, many Chinese groups have dedicated their efforts on the ANAMMOX research. The highest nitrogen removal rate in laboratory scale has been obtained from a research group in China which is a strong evidence of development in ANAMMOX research. Three new species of ANAMMOX bacteria have been identified, among them two species are responsible for simultaneous ammonium and sulfate removal which is a new addition in ANAMMOX process for simultaneous treatment. Pilot-scale operations are expanding and using domestic expertise, technology, manpower, and so forth, full scale application with domestic expertise and technology is beckoning for commencement. Overall China's progress in ANAMMOX process is appreciating and time befitting.

### 6.1. Highlights

Discovery of ANAMMOX and sulfate-dependent ANAMMOX bacteria, ANAMMOX process and sulfate-dependent ANAMMOX process development for simultaneous removal, and pilot scale to full scale ANAMMOX process application were reviewed.

## Figures and Tables

**Table 1:** Natural distribution, abundance, and identification of ANAMMOX bacteria from different ecosystems in China.

Ecosystem	Abundance	ANAMMOX Bacteria	Area	Reference
South China Sea, Tai Lam Chung Water Reservoir and Mai Po Nature Reserve	N.A.	Scalindua, Jettenia, Kuenenia, Anammoxoglobus, and Brocadia	South China	[53]
South China Sea	1.19 × 10^4^ to 7.17 × 10^4^	Scalindua	South China	[54]
Jiaozhou Bay	3.89 × 10^1^ to 3.89 × 10^6^	Scalindua, Jettenia, Brocadia, Anammoxoglobus, and Kuenenia	East China	[55]
Shengli Oilfield at Yellow River Delta	4.4 × 10^6^ copies/g of soil	Brocadia, Kuenenia, Scalindua, Jettenia, and Anammoxoglobus	East China	[56]
Paddy field	N.A.	Kuenenia, Anammoxoglobus, Jettenia, and Brocadia	North China	[57]
Freshwater sediments of the Xinyi River	N.A.	Scalindua	East China	[58]
Different natural ecosystems	N.A.	Brocadia, Kuenenia, Scalindua, and Jettenia	East China	[12]
Pharmaceutical wastewater	N.A.	Photobacterium phosphoreum	East China	[40]
Monosodium glutamate (MSG) wastewater	N.A.	Kuenenia	East China	[59]
Qiantang River	N.A.	Brocadia, Kuenenia and Scalindua	East China	[13]
Honghe State Farm soil	N.A.	Scalindua	Northeast China	[60]
Wetland	10^5^ copies/g	Scalindua, Kuenenia, Brocadia, and Jettenia	South China	[61]
Qiantang River	N.A.	AOA and AOB	East China	[62]
Paddy soil	N.A.	Anammoxoglobus, Jettenia, and Anammoxoglobus propionicus	East China	[63]
Qiantang River	N.A.	Brocadia, Kuenenia and Scalindua	East China	[64]
Paddy Soil Column	N.A.	Nitrososphaera, Nitrosotalea and Nitrosopumilus	East China	[65]
Jiaojiang Estuary	N.A.	Brocadia, Kuenenia, Scalindua, and Jettenia	East China	[66]
Agricultural soils	6.38 ± 0.42 × 10^4^ to 3.69 ± 0.25 × 10^6^	Brocadia, Kuenenia, Jettenia, and Anammoxoglobus	East China	[67]
Mai Po Nature Reserve	N.A.	Scalindua, Kuenenia, Scalindua, and Anammoxoglobus	South China	[68]
Paddy soil	6.5 × 10^3^ to 7.5 × 10^4^	Brocadia and Jettenia	North China	[69]
Waste Lake	N.A.	Brocadia, Kuinenia, and Scalindua	East China	[70]

N.A.: not available; AOA: ammonia-oxidizing archaea, AOB: ammonia-oxidizing bacteria.

**Table 2:** Overview of high ANAMMOX performance in China.

Reactor	HRT	Influent conc. mg/L		Removal efficiency (%)		NLR kg-N/m^3^/d	NRR kg-N/m^3^/d	Reference
		NH_4_–N	NO_2_–N	NH_4_–N	NO_2_–N			
UASB reactor	0.2 h	300	360	90	N.A.	89.1	74.3–76.7	[28]
EGSB reactor	6–0.3 h	494	522	94.68	99.84	77.84	57.14	[29]
EGSB reactor	1.5 h	661.9	767.2	71.7	94.1	22.87	18.65	[30]
AGSB reactor	1.1 h	400	500	N.A.	N.A.	N.A.	15.40	[26]
UASB reactor	N.A.	320–340	350–380	96.1	95.2	7.2	6.2	[27]
Upflow filter system	1.99 h	305	304	74.2	92.4	7.34	6.11	[71]
UASB reactor	0.12 h	16.87 ± 2.09	20.57 ± 2.31	92.81	94.35	N.A.	5.72	[72]
ALR reactor	5.4 h	546	N.A.	94.4	N.A.	2.37	2.29	[73]
UASB reactor	0.28 h	16.87 ± 2.09	20.57 ± 2.31	78.45	92.31	N.A.	2.28	[72]
UBF reactor	1.54 h	976.0	1280	88.84	98.1	34.5	N.A.	[74]
SBR reactor	0.18 d	500	580	97	97	0.156	N.A.	[75]
SBR reactor	3 d	268	345	83.6	100	N.A.	N.A.	[76]
SBR reactor	1.5 h	N.A.	N.A.	80.9	88	N.A.	N.A.	[77]

N.A.: not available.

**Table 3:** Overview of the sulfate-dependent ANAMMOX process investigation in China.

Source	pH	Reactor	VSS g/L	HRT	Influent mg/L		Removal ratio	Average removal efficiency (%)		Reference
					NH_4_–N	SO_4_–S	NH_4_ : SO_4_	NH_4_–N	SO_4_–S	
K_2_SO_4_	8.5	Lab-scale reactor	N.A.	1 d	229	163	2.01 : 1	44.4	40	[15]
NH_4_Cl										

NaNO_2_	8–8.2	NRBC reactor	0.32–0.054	4–24 h	288^b^	N.A.	1.71 : 1.75	50^b^	N.A.	[14]
(NH_4_)_2_SO_4_										
(NH_4_)_2_SO_4_										

NH_4_Cl	7.5–8.5	UASB reactor	N.A.	1.5 d	60	240	2 : 1	40	30	[38]
NaNO_2_										
NaSO_4_										

(NH_4_)_2_SO_4_	7.5	Expanded bed reactor	14.9	1 d	843	130	2 : 1	56.82^a^	71.67^a^	[37]
NaNO_2_										

Na_2_S·9H_2_O	8.33 ± 0.18	UASB reactor	35.7	0.8 h	350	264	N.A.	94.7	N.A.	[39]

^a^Concentration in mg/L, ^b^concentration in mmol/L/D, N.A.: not Available.

**Table 4:** Brief description of pilot-scale application of ANAMMOX process in China.

Wastewater	Reactor	Working volume	VSS g/L	pH	Temp °C	Influent mg/L		Ratio	Removal efficiency (%)		NRR kg/m^3^/d	Reference
						NH_4_–N	NO_2_–N	NH_4_ : NO_2_	NH_4_–N	NO_2_–N		
Synthetic wastewater	UASB	2.5 m^3^	43.5	6.8	5–27	299	336	1 : 1.12	84	98	1.30	[119]
Synthetic wastewater	UASB reactor	50 L	3.78	7.5–8.0	37	448	575	1 : 1 ± 0.26	93	99	27.8	[78]
DAFW	UASB reactor	68 L	2.2	6.8–7.0	35 ± 1	150–180	120–150	1 : 1.26	85	90	N.A.	[117]
Synthetic wastewater	UASB reactor	20 L	7.2	7.5–8.0	35	377	557	1 : 1	85.5	91.4	640 TN	[120]
PW	MABR reactor	22.5 m^3^	10	7.2–8.5	12–26	N.A.	N.A.	N.A.	98	N.A.	N.A.	[42]

DAFW: dry-spun acrylic fiber wastewater, PW: pharmaceutical waste, N.A.: not available.

**Table 5:** Brief description of full scale ANAMMOX plants in China.

Company/Institution	Area	Substrate	Reactor volume m^3^	Designed load kg-N/d	Year	Reference
Zhejiang University	Zhejiang	Pharmaceutical waste	10	5	2010	[40]
Zhejiang University	Zhejiang	Monosodium glutamate wastewater	60	5	2008	Personal communication
National Chiao Tung University	Taiwan	Landfill leachate	384	304 m^3^/d*	2006	[43]

Angel Yeast	Yichang	Yeast production	500	1000	2009	Paque [44]
Meihua I	Tongliao	Monosodium glutamate (MSG)	6600	11000	2009	
Meihua II	Tongliao	MSG	4100	9000	2010	
Shandong Xiangrui	Shandong	Corn starch and MSG	4300	6090	2011	
Jiangsu Hangguang Bio-engineering	Wuxi	Sweetener	1600	2180	2011	
Xinjiang Meihua Amino Acid	Wujiaqu	MSG	5400	10710	2011	
Kuaijishan hoaxing Winery	Shaoxing	Distillery	560	900	2011	

*Average leachate flow of 304 m^3^/d with a sludge retention time between 12 and 18 d.
